# Use of target drift in heading judgments

**DOI:** 10.1167/jov.25.7.9

**Published:** 2025-06-20

**Authors:** Li Li, Simon K. Rushton, Rongrong Chen, Jing Chen

**Affiliations:** 1 Faculty of Arts and Science, New York University Shanghai, Shanghai, China; 2 NYU-ECNU Institute of Brain and Cognitive Science, New York University Shanghai, Shanghai, China; 3 Department of Psychology, The University of Hong Kong, Hong Kong SAR; 4 School of Psychology, Cardiff University, Tower Building, Park Place, Cardiff, Wales, UK; 5 Faculty of Science & Technology, Guangdong Provincial/Zhuhai Key Laboratory IRADS, Beijing Normal-Hong Kong Baptist University, Zhuhai, China

**Keywords:** heading, target drift, optic flow, egocentric direction, locomotion

## Abstract

The change in direction of a target object relative to a translating observer (or a point fixed relative to the observer), “target drift,” provides information about the observer's direction of self-movement (i.e., heading) with respect to the target. Relative drift rate (normalized with cues to motion-in-depth) provides information about the observer's absolute direction of heading relative to the surrounding scene. We investigated the utility of target drift by comparing heading judgments with target drift and “extra-drift” cues (the cues available in the changing optic array except target drift) in isolation and together during simulated forward translation. Across four experiments, we found that with the target drift cue alone, participants were able to make precise judgments of both nominal and absolute heading (≤1.53°). Judgments were at least as precise with the target drift cue alone as with extra-drift cues alone. The addition of extra-drift cues to the drift cue did not improve precision, and the pattern of reaction times suggests that the two cues are processed independently. We conclude that target drift can be an effective and powerful cue for heading judgments.

## Introduction

When we move through a real or virtual environment, we can easily report whether we are on a course to the left or right of objects or features in the environment. What cue provides the most useful information to accomplish this task? The standard answer is optic flow. Optic flow is the pattern of optical motion available at the eye that results from the movement of the observer relative to objects in the scene (Gibson, 1950). When we travel on a straight path (translation), the optic flow field forms a radial pattern. The point from which the motion radiates in this radial pattern is known as the focus of expansion (FoE; Calvert, 1950; Gibson, 1950; see also Grindley, 1942, as discussed by Mollon, 1997 and Niehorster, 2021) and indicates the direction of translation, or “heading.” It has been shown that observers can judge, from optic flow alone, whether they are heading to the left or right of a target object with the precision of about 1° (Crowell & Banks, 1993; van den Berg, 1992; Warren, Morris, & Kalish, 1988).

In addition to global optic flow, there are a number of alternative or complimentary visual cues in the changing optic array that can also be used to judge heading direction. The cue of particular interest here is target drift. Before describing this cue, we provide a brief summary of other visual cues to heading direction.

**Figure 1.**
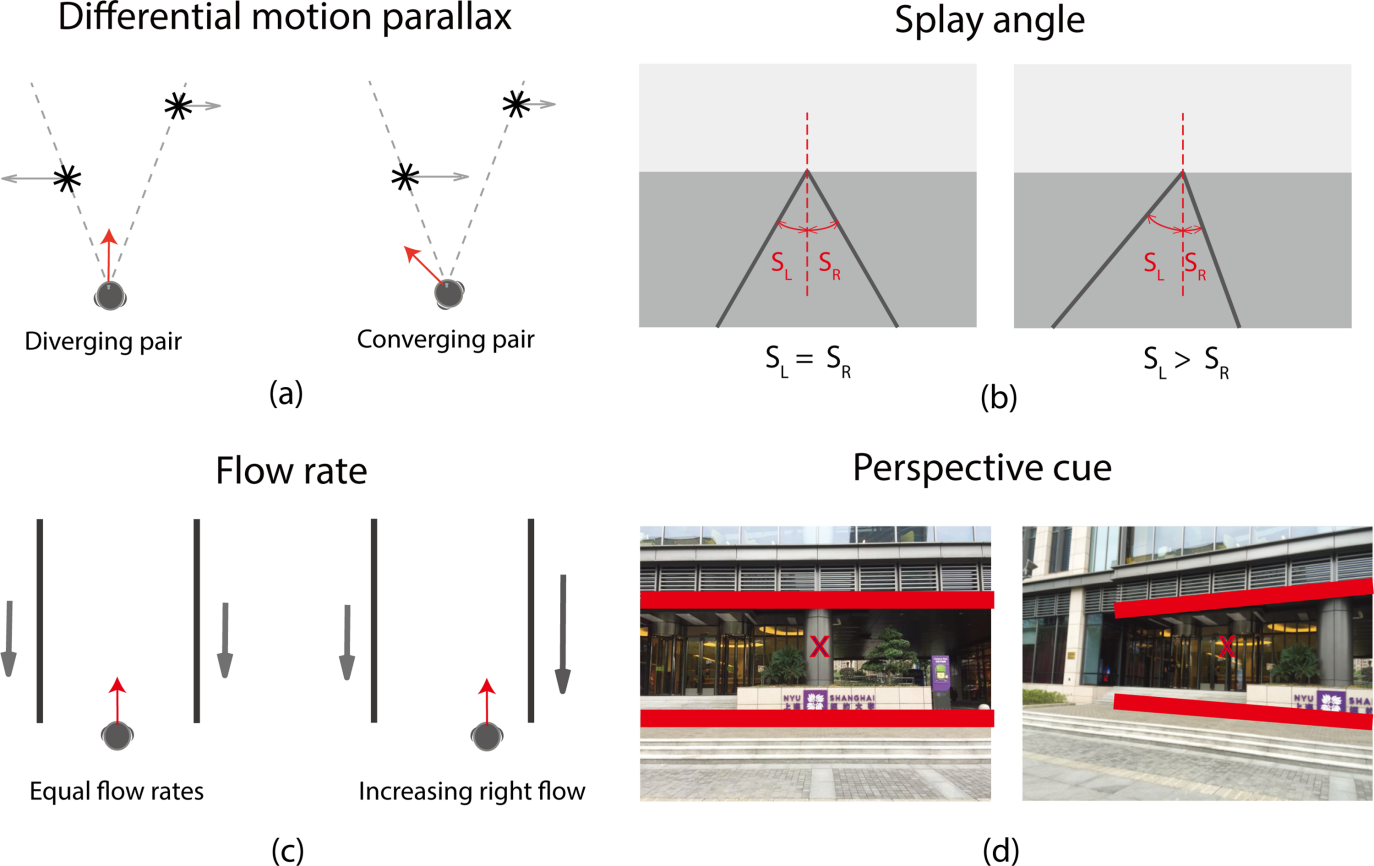
Illustrations of alternative visual cues for heading judgments. (**a**) Differential motion parallax. The heading direction (red arrow) is positioned between pairs of diverging objects (left panel: the near object moves in the opposite direction and at a faster speed than the far object, leading to pairs of diverging objects) and away from pairs of converging objects (right panel: the near object moves in the same direction but at a faster speed than the far object, leading to pairs of converging objects). (**b**) Splay angle. Equal and opposite splay angles indicate a centered position on the path (left panel) and an increase in left splay angle indicates a drift to the right (right panel). (**c**) Flow rate. Equal flow rates indicate centered movement within a corridor (left panel) and a faster flow rate on the right-side signals movement closer to that side (right panel). (**d**) Perspective cue. In scenes with large vertical surfaces, the direction of heading (red “x”) can be inferred from changes in perspective shape. Here a shift in heading from center (left panel) to the left (right panel) transforms the shape of a vertical surface from rectangular to trapezoidal.

When the scene contains distinct objects, the relative motion of objects in the retinal image, differential motion parallax, provides information about the direction of heading (Cutting, Springer, Braren, & Johnson, 1992). Specifically, the direction of heading is typically located between pairs of objects that are diverging in the retinal array and away from pairs of converging objects (Figure 1a), and the combination of relative motion information from multiple pairs of objects provides probabilistic information about the exact direction of heading (Wang & Cutting, 1999).

When we travel along a path, corridor, or road, splay angle (i.e., the angle between the optical projection of the road edge and a vertical line in the image plane), the relative speed of optic flow on two sides, and perspective shape, are also informative about location and heading.

The relative splay angles of the left and right sides provide information about lateral location on the path (Figure 1b). If they are equal in magnitude and opposite in direction, we are in the middle of the path. If they remain constant then lateral position remains constant. Changes in splay angle indicate changes in lateral position. If the left splay angle increases and the right splay angle decreases, we are drifting rightward and vice versa (Beall & Loomis, 1996; Li & Chen, 2010).

The relative flow rates on the left and right sides also provide information about lateral position. When moving along a corridor, if the rates are equal, we are traveling down the middle (Duchon & Warren, 2002; Srinivasan, Lehrer, Kirchner, & Zhang, 1991). If the rates remain constant, then lateral position remains constant. If the flow rate increases on the right, then we are drifting rightward and vice versa (Figure 1c). Relative flow rate is effectively a crude version of the optic flow cue.

When we are heading toward an internal or external corner, the relative size of the two walls in the perspective projection provides information about our location relative to the corner, and changes in relative size provide information about the direction of heading (Beusmans, 1998). This can be generalized to the cases when there are large vertical planar surfaces visible in the scene, heading can be derived from the change in perspective shape (Figure 1d).

In summary, in environments with clearly defined surfaces and edges, splay angle, relative flow rate, and perspective shape are all potentially powerful cues to the direction of heading.

During natural locomotion (walking, running, & biking etc.), the direction of the target object relative to the body (target egocentric direction) also provides information about the direction of heading, e.g., when a target is to our left and we walk straight forward, we expect to head to the right of the target and vice versa (Rushton, Harris, Lloyd, & Wann, 1998). If the egocentric direction is held constant, then we are on a straight or low equiangular spiral path to the target.

Target drift (Llewellyn, 1971; Rock, 1966), the focus of this article, is the change of target egocentric direction (or the change of direction relative to a point that is fixed relative to the observer). This cue is sufficient for an observer to make nominal judgments about whether they are heading to the left or right of a target object (e.g., if a target drifts rightward, the observer is heading to the left of the target and vice versa).

**Figure 2.**
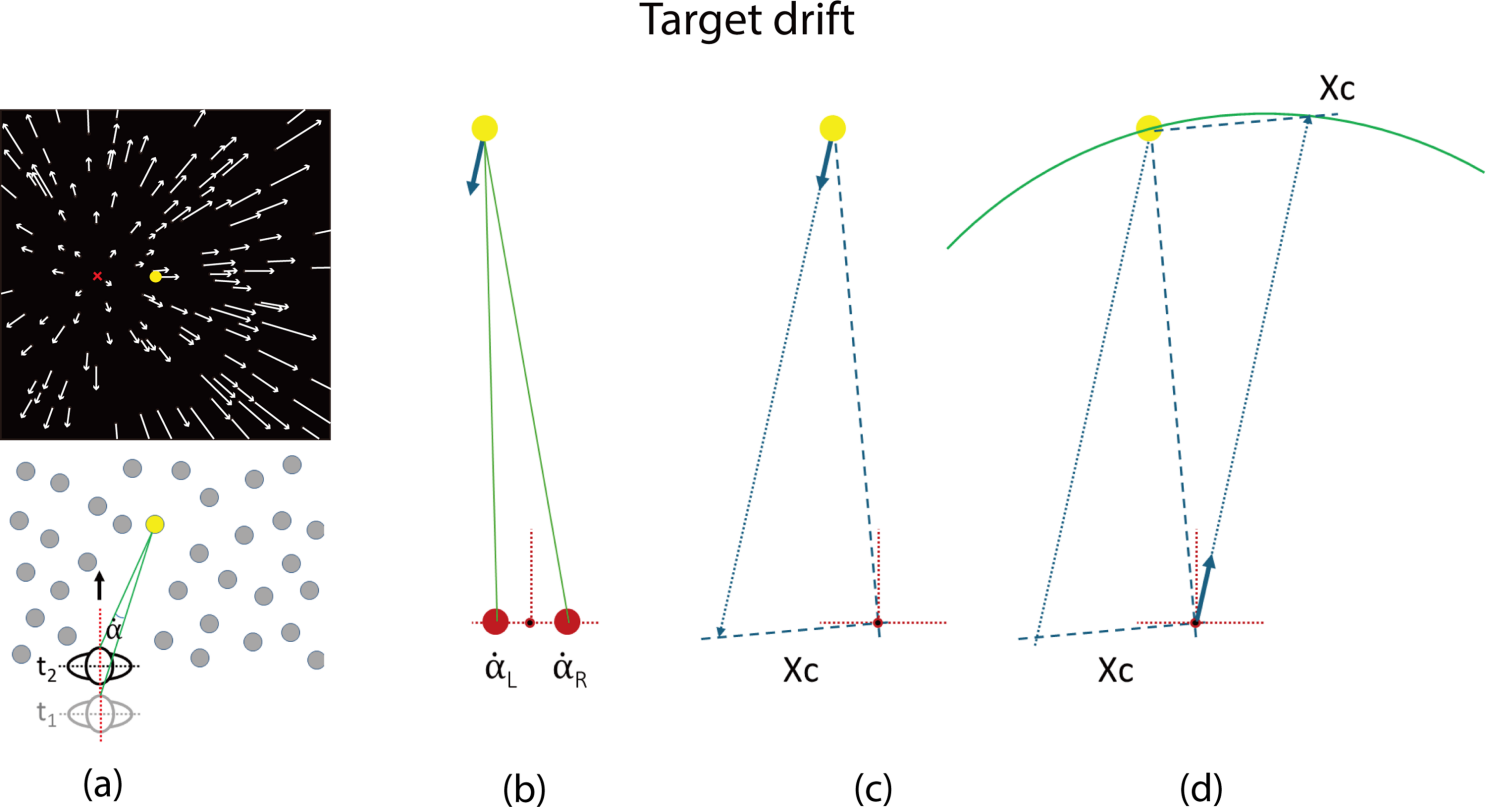
Illustrations of how target drift can be used for heading judgments. (**a**) An illustration of optic flow and target drift considered from the Cyclopean eye (mid-point of the left and right eyes). The top panel shows the frontal view of a flow field generated when an observer translates through a 3D random dot cloud. The movement of any element in the flow field can be regarded as a drift cue. Here we highlight a target object (the yellow dot) to illustrate that the observer's heading direction is to the left of a target in the scene when it drifts rightward. The motion vectors (indicated by the white lines with arrowheads) stream out from the FoE (indicated by the red “x”), which is to the left of the yellow target. Therefore, optic flow also indicates that the observer is heading to the left of the target. The bottom panel shows the bird-eye view and the target drift rate ($\dot{\alpha }$) from time point_1_ to t_2_. (**b**) An illustration of left and right eye (red filled circles) observing the target that is on a course to pass to the left of the eyes. For ease of comparison with the background literature on perception of motion in depth, the target is seen as moving toward the observer (blue arrow) rather than vice versa. (**c**) Xc (given by $\dot{\alpha }$_CYC_/$\dot{\alpha }$_Disp_, specified in multiples of the interocular separation) is the distance at which the target will pass the observer measured in the plane that contains the Cyclopean eye and is perpendicular to the line of sight. (**d**) Following reflection around the line of sight, the blue arrow now indicates movement of the observer. It can be seen that Xc is also the distance at which the observer will pass the target measured in the plane that contains the target and is perpendicular to the line of sight. The green line indicates the arc along which a probe is placed to indicate the judged direction of heading.

It is useful to clarify the relationship between target drift and optic flow. Consider the motion information available to a translating observer wishing to intercept or avoid an object in the scene, such as the target object shown by the yellow dot in Figure 2a. Optic flow is the motion of all elements in the scene, *relative to each other*. Target drift is the motion of a single element (such as the yellow target) in the flow field *relative to the observer (or a point that is fixed relative to the observer)*.

Target drift rate provides probabilistic information about how far from the target the observer is heading. For judgments of the absolute direction of heading relative to the scene, a probabilistic cue is not sufficient. It is necessary to normalize target drift rate to recover the exact heading direction.^1^ In natural environments, information to normalize target drift rate and estimate the absolute heading direction is available, and this information is the disparity in drift rates. Specifically, the target is viewed from two points of observation, the left and right eyes, producing slightly different two drift signals, $\dot{\alpha }$_L_ and $\dot{\alpha }$_R_ (see Figure 2b). If we define the Cyclopean target drift, $\dot{\alpha }$_CYC_, as $\dot{\alpha }$_CYC_ = ($\dot{\alpha }$_L_ + $\dot{\alpha }$_R_)/2, and the disparity in target drift rates of the two eyes, $\dot{Ø}$, as $\dot{Ø}$ = ($\dot{\alpha }$_L_ + $\dot{\alpha }$_R_), then $\dot{\alpha }$_CYC_ / $\dot{Ø}$ defines the distance Xc at which the target will pass the observer measured in the plane that contains the Cyclopean eye and is perpendicular to the line of sight (Regan & Kaushal, 1994), where the unit of distance is the separation between the eyes (i.e., the interocular distance, see Figure 2c). It can be seen from Figure 2d that by reflection about the line of sight, Xc is also the distance at which the observer will pass the target if they continue along their current trajectory. Xc can also be obtained by use of looming rate (rate of change of optical size of the target) when the size of the target is known (see Regan & Gray, 2000; Regan & Kaushal, 1994; Rushton & Duke, 2007; Duke & Rushton, 2012 for the theoretical background) or from the ratio $\dot{\alpha }$_L_ $\dot{\alpha }$_R_ (see Beverley & Regan, 1973).

Target drift could be of particular importance during vehicular or assisted locomotion, when there is no direct mapping between the direction of locomotion and the orientation of the body (i.e., the relationship between the locomotor axis and the egocentric straight ahead is unknown). Despite the considerable body of work investigating the use of visual cues in heading judgments, there has been very little research on the observer's sensitivity to the target drift cue since the original work by Llewellyn (1971). This is mainly because target drift has been deliberately excluded so that researchers could study other cues in isolation. Target drift is typically removed by only showing a target object at the end of the stimulus display, by placing the target on the horizon to eliminate target drift, by adding simulated gaze rotation in the display to remove or confound target drift, or by asking observers to make self-movement judgments relative to a reference axis (such as the straight ahead) in the egocentric space (e.g., Fetsch, Pouget, DeAngelis, & Angelaki, 2011; Foulkes, Rushton, & Warren, 2013a; Foulkes, Rushton, & Warren, 2013b; Li, Chen, & Peng, 2009; Li & Cheng, 2011; Li, Sweet, & Stone, 2006; Royden, Banks, & Crowell, 1992; Stone & Perrone, 1997). One notable exception is Experiment 1 in the study by Wilkie and Wann (2003). In their experiment, the target drift cue (what they called the “extra-retinal” cue) was placed in conflict with optic flow, and the results pointed to the use of a combination of flow and target drift cues in heading judgments.

In the current study, we evaluated target drift as a cue to heading judgments during self-movement. In Experiments 1–3, following the seminal work by Warren and colleagues (Warren & Hannon, 1988; Warren, Morris, & Kalish, 1988) and subsequent or parallel work by many other laboratories (e.g., Cutting et al., 1992; Macuga, Loomis, Beall, & Kelly, 2006; Royden, Crowell, & Banks, 1994; van den Berg, 1992), we asked observers to make judgments of whether they were heading to the left or right of a target object. We varied the angle between the target and the direction of heading (i.e., target-heading angle) at the beginning of the trial in the range of ±2.5° and quantified how large the angle needed to be for observers to correctly judge whether their heading was to the left or right of the target 75% of the time at the end of the trial. In Experiment 4, following several other researchers and our previous studies (e.g., Banks, Ehrlich, Backus, & Crowell, 1996; Li & Warren, 2000; Li & Warren, 2004; Li, Peli, & Warren, 2002), instead of asking observers to make nominal left/right judgments of heading relative to a target, we adopted the method of adjustment and asked observers to make judgments of absolute heading relative to the scene at the end of the trial. We also increased the range of tested initial target-heading angles up to 10°. The deviation angle between the judged and the actual target-heading angle was calculated as heading error indicating the accuracy of heading judgments.

Across all four experiments we found that the precision of heading judgments was at least as high with the target drift cue alone as with the *extra-drift* cues (all the cues in the changing optic array except target drift). We also found mixed evidence of the combination of both drift and extra-drift cues in heading judgments. In the General Discussion, we review the findings of the current study and consider how the salience and, hence, usefulness of different visual cues for heading judgments vary as a function of the characteristics of the scene and the task performed. We then discuss what the results of the four experiments in the current study tell us about the use of the target drift and extra-drift cues for heading judgments.

## Experiment 1: Judgments of heading relative to a target

### Methods

#### Participants

Twenty students and staff (nine males, 11 females; 18 naïve as to the purpose of the experiment) between the age of 19 and 43 (average age = 26) at The University of Hong Kong participated in the experiment. All had normal or corrected to normal vision and provided informed consent. The experiment was approved by the Human Research Ethics Committee for Non-Clinical Faculties at The University of Hong Kong. We determined the sample size using power analyses (with power set at 0.8) based on the observed effect sizes from previous similar studies (e.g., Rushton, Chen, & Li, 2018).

#### Visual stimuli

The visual display (56°H × 33°V, 120 Hz, viewing distance: 56.5 cm) simulated forward self-movement at a typical walking speed of 1 m/s in a three-dimensional (3D) scene (depth range: 4.4–8.4 m). 8.4 m was chosen as the far distance because most manmade environments such as rooms and corridors do not extend much further than this distance. We chose to place objects no closer than 4.4 m because once objects approach personal space, mechanisms concerned with interaction (foot placement relative to the object, planning reaching movements etc.) would likely come into play. Under natural circumstances, with scene objects in a range of 4.4–8.4 m, stereo disparity cues would provide information about distance and depth. Therefore we rendered the scene in stereo.

It is difficult for a participant to fuse large uncrossed disparities on a standard monitor at arm's reach because of the mismatch with the accommodative or focus cues provided by the surface of the monitor. Therefore we scaled the scene dimensions and the self-movement speed down by a factor of 8. Consequently, the values used to construct the stimuli were a scene-movement speed of 0.125 m/s and a depth range of 0.55 to 1.05 m. The scaling reduced the conflict between the vergence demand and the accommodative demand for near and far objects but kept the angular velocities consistent with those that would be experienced in a 4.4–8.4 m scene approached at 1 m/s.

Note, because of the inclusion of stereo cues, each eye picks up slightly different flow fields and target drift cues. The differences between the left and right flow fields provide additional information that could improve the precision of heading judgments in the presence of noise (e.g., van den Berg & Brenner, 1994, but see Rushton, Harris, & Wann, 1999). The slight differences between target drift rates at the two eyes, $\dot{\alpha }$_L_ and $\dot{\alpha }$_R_, in conjunction with the knowledge about the separation of the two eyes, provide information about the exact heading direction in the scene (as explained in the Introduction).

**Figure 3.**
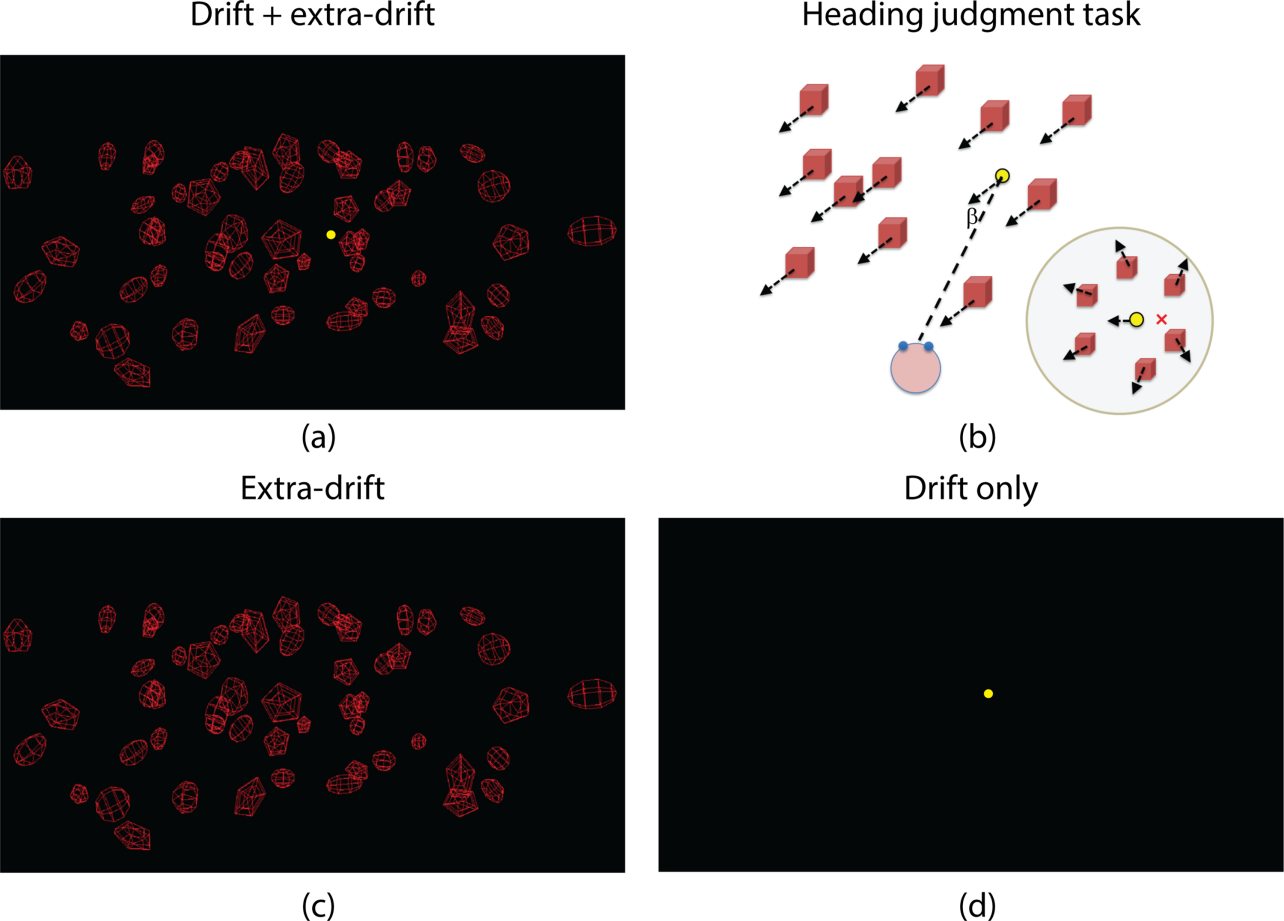
Illustrations of visual stimuli. Stimuli contained disparity depth cues that are not shown here. (**a**) The drift + extra-drift condition. The display composed of an array of 55 red wireframe scene objects of randomized size and orientation located at randomized distances ahead of the observer. The target sphere was rendered in yellow. The display was rendered with disparity cues to depth and the observer viewed the scene through shutter glasses. (**b**) The heading judgment task. Schematic diagram. Dashed line shows the initial direction of the target sphere relative to the observer at the beginning of the trial. Arrows show the (common) direction of movement of all scene objects, including the target sphere. β indicates the target-heading angle. Inset shows observer's view with the direction of heading indicated by the red “x”. Here the target sphere is drifting to the left of the observer; therefore the direction of heading of the observer is to the right of the target sphere. (**c**) The extra-drift condition. This display is identical to the display in the drift + extra-drift condition except that the target sphere only appeared at the end of the trial when the motion stopped and thus could not provide the target drift cue. (**d**) The drift-only condition. All scene objects were removed, and the scene contained only the yellow target sphere.

Three display conditions were tested. Figure 3a illustrates the display used in the *drift + extra-drift* condition. The scene consisted of 55 wireframe objects that moved in a common 3D direction to simulate forward self-movement. Each scene object was composed of 5 “slices” and 5 “stacks” (see “gluSphere” in OpenGL handbook), had an initial radius of 1 cm, and was placed on a regular 11 × 5 grid before its position was randomly perturbed (±1 cm horizontal, ±1.2 cm vertical, and ±25 cm in depth) to produce an array of objects that were randomly distributed but not overlapping. The target object (shown in yellow) was a sphere of 0.1 cm radius whose movement provided a target drift cue. The initial position of the target was 3.5 cm (equivalent to 28 cm in the full-scale environment) to the left or right of the middle of the array (depth: 0.8 m, equivalent to 6.4 m in the full-scale environment) and replaced the scene object in that location. From the viewpoint of the participant, the initial direction of the target was 2.5° to the left or right of the straight ahead (which was aligned with the vertical midline of the display).

Figure 3b illustrates the heading judgment task. The yellow target sphere moved with the same velocity as the other scene objects and was drifting to the left of the observer. Accordingly, the heading direction of the observer relative to the environment was to the right of the target sphere. Figure 3c illustrates the display used in the *extra-drift* condition. It was identical to the display used in the drift + extra-drift condition except that the yellow target sphere only appeared at the end of the trial when the motion stopped and thus did not provide the target drift cue (i.e, the target position was exactly the same as in the drift + extra-drift condition except that the target only appeared at the end of the trial). Figure 3d illustrates the display used in the *drift-only* condition. All background scene objects were removed and the scene contained only the yellow target sphere.

We used the method of constant stimuli to measure the precision of the heading judgment performance. We selected 15 equally spaced initial target-heading angles (β) with the heading direction ranging from −2.5° (left) to 2.5° (right) of the target at the beginning of the trial (Figure 3b).

#### Equipment

Anti-aliased stimuli were rendered, using OpenGL, on an nVidia Quadro K2000 graphics card and displayed on an Asus VG278H 27” LCD monitor at a resolution of 1920 × 1080 at 120 Hz (60 Hz per eye). With their heads stabilized by a chin rest at the viewing distance of 56.5 cm in a dark room, participants viewed the stimuli through a pair of LCD shutter glasses (Nvidia 3D Vision 2) driven by an infrared emitter built into the monitor. The left and right eye images were temporally interleaved and displayed in synchrony with the opening and closing of the left and right eye shutter glass lenses to create a stereoscopic presentation.

#### Procedure

On each trial, a static view of the scene appeared for one second to allow participants to fuse the stereo half-images into a 3D scene. The objects then moved for 1.5 seconds to simulate forward movement of the observer. At the end of the simulated movement for all three display conditions, a black blank screen with the yellow target object appeared, and participants pressed a mouse button to indicate whether they had been heading to the left or right of the target.

Each participant completed 120 trials (8 trials × 15 levels of target-heading angles) in a random order for each display condition. Participants received 10 randomly selected training trials at the beginning of each display condition. No feedback was given on any trial. The testing order of the three display conditions was counterbalanced between participants. An experiment session typically lasted 30 minutes.

#### Data analysis

Given that the target-heading angle increased during the course of the trial and heading judgments were made at the end of the trial, we first computed the final target-heading angles and then plotted the proportion of rightward judgments against final target-heading angle for each participant. We then fitted a cumulative Gaussian function to the data. We obtained the standard deviation (SD) of the fitted Gaussian function, which is inversely related to the slope of the fitted curve, as the measure of the precision of heading judgments. The smaller the SD, the higher the precision.

### Results

**Figure 4.**
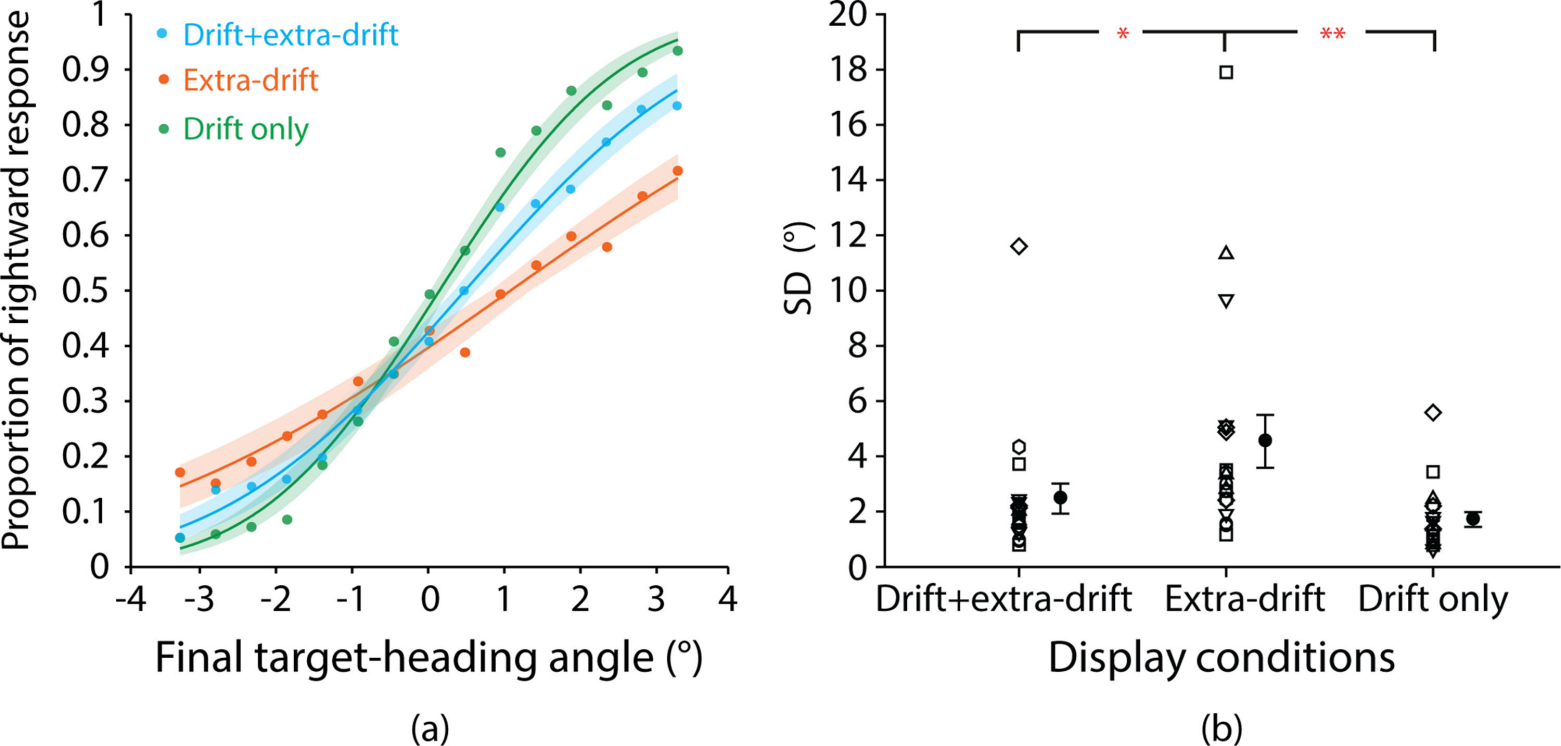
Heading judgment data for the three display conditions. (**a**) Proportion of “rightward” judgments (circles) as a function of final target-heading angle for the composite observer. Data were fitted with a cumulative Gaussian function (solid lines). (**b**) SD of the fitted Gaussian curve for each individual participant along with the group mean for the three display conditions. Lower SDs indicate higher precision. Error bars are ±1 SE across 19 participants. *p < 0.05; **p < 0.01.

One participant (female) showed a random pattern of heading judgments that could not be fitted by a cumulative Gaussian function for all display conditions. This participant's data were excluded from the data analysis. For illustrative purposes, for each display condition, we combined the data across the remaining 19 participants to create a “composite observer” and fitted it with a cumulative Gaussian function (Figure 4a). The slope of the fitted curve was steeper for the drift + extra-drift and the drift-only conditions than the extra-drift condition, indicating that participants were able to make more precise heading judgments when the display contained the target drift cue than when not.

Figure 4b plots SD of the fitted Gaussian curve for each individual participant along with the group mean for the three display conditions. The lower the SD, the higher the precision of heading judgments. We conducted a one-way repeated-measures analysis of variance (ANOVA) to test the statistical significance of any differences. We checked sphericity using Mauchly's test. If sphericity was violated, we reported Greenhouse-Geisser corrected degrees of freedom and *p*-values when *ε* < 0.75, or Huynh-Feldt corrected values when *ε* > 0.75.

Precision showed a clear difference between display conditions (main effect of display condition: *F*(1.14, 20.46) = 6.86, *p* = 0.014, *η^2^* = 0.28). Newman-Keuls tests showed that SD was significantly lower (precision higher) in the drift-only (mean SD ± SE: 1.72° ± 0.27°) than the extra-drift condition (4.55° ± 0.96°, *p* = 0.0029). The addition of target drift to extra-drift cues also produced a significant decrease in SD (increase in precision) in the drift + extra-drift condition (2.47° ± 0.55°) compared with the extra-drift condition (*p* = 0.013).

We noted a couple of outliers in each display condition in Figure 3b. To test the robustness of the findings, we repeated the analysis using nonparametric sign tests and found the same pattern of significance to the parametric tests. The pattern of medians (drift + extra-drift: 2.00°; extra-drift: 2.98°; drift-only: 1.37°) also mirrored that of the means.

### Discussion

The precision of heading judgments is higher with the target drift cue alone than with extra-drift cues alone. The addition of target drift to extra-drift cues also produces a significant increase in precision compared with extra-drift cues alone. Because precision is not higher with both cues present than with the target drift cue alone, this does not support an early optimal combination of the target drift and extra-drift cues for heading judgments.

In the drift-only condition, the target object was visible at all times. In the extra-drift condition, the target was only visible at the end of the trial. It could be argued that in the extra-drift condition, participants had to remember their heading during the trial, and the need to use memory reduced the precision of heading judgments at the end of the trial. Data from Warren et al., (1988) speak to this issue. In their first experiment, the authors measured the precision of heading judgments using optic flow stimuli that had a target line visible on the horizon throughout the trial. In a subsequent experiment, the target line was only presented after the optic flow at the end of the trial. Participants were asked to judge their heading relative to the target line (as in the current experiment). They found that the precision of judgments in the two experiments was very similar.

Furthermore, we can address the memory issue by comparing the precision of judgments in the drift-only and the drift + extra-drift conditions. Because the target was visible throughout the trial in both conditions, there were no differences in memory requirements. What differentiates the two conditions is the presence of extra-drift cues. If extra-drift cues are important, adding them to the display should make judgments more precise. The fact that precision is similar in the combined cue condition and the drift-only condition thus does not support a memory-based explanation of the lower precision in the extra-drift condition.

The duration of the motion stimulus (1.5 s) was fixed and relatively long in this experiment. One question arises is whether the relative informativeness of the two types of cues varies with stimulus duration. This is plausible because different motion cues may become more or less effective over time because of temporal integration processes in the visual system. For instance, target drift relies on tracking target visual direction over time, which may become more reliable with longer stimulus durations, whereas some of extra-drift cues are immediately salient thus do not benefit as much from extended exposure. If such differences exist, the stimulus duration we chose in the current experiment might have advantaged the target drift cue over extra-drift cues. To examine this possibility, we designed the next experiment to systematically test whether cue effectiveness depends on stimulus duration.

## Experiment 2: Varying stimulus duration

In this experiment, we investigated how the precision of heading judgments evolved over time. We varied the stimulus duration from 0.2–1.6 seconds.

### Methods

#### Participants

Twelve participants (six males, six females; all naïve as to the purpose of the experiment) between the age of 18 and 36 (average age = 27) at the University of Hong Kong participated in this experiment. Four of these participants also participated in Experiment 1. All had normal or corrected to normal vision and provided informed consent. The experiment was approved by the Human Research Ethics Committee for Non-Clinical Faculties at The University of Hong Kong. Although the experiments in this article are presented in a logical order, Experiment 2 was in fact run second to last, which allowed us to use the observed effective size in previous experiments to reduce the sample size.

#### Visual stimuli and procedure

All three display conditions of Experiment 1 were tested. The displays were identical to those in Experiment 1 except that the duration of the simulated self-movement was changed from 1.5 seconds in Experiment 1 to 0.2 second, 0.4 second, 0.8 second, or 1.6 seconds.^2^

The testing procedure was the same as in Experiment 1. Each participant completed 480 trials (8 trials × 15 levels of target-heading angles × 4 stimulus durations) in a random order for each display condition. Participants were asked to take a short five-minute break after each display condition. The testing order of the three display conditions was counterbalanced among participants. An experimental session, with breaks, lasted around two hours in total.

### Results

**Figure 5.**
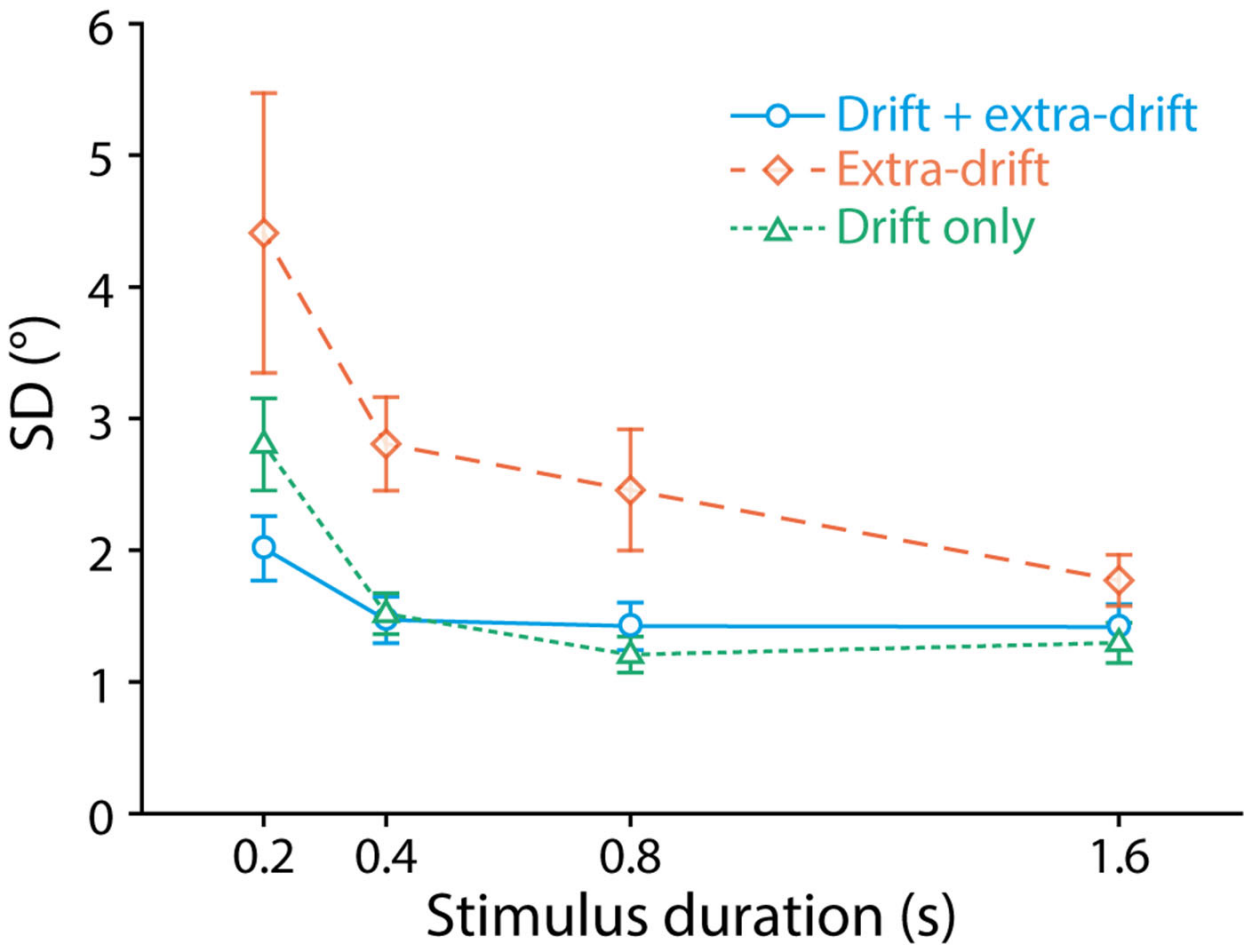
Mean SD of the fitted Gaussian curve as a function of stimulus duration for the three display conditions. Lower SD indicates higher precision. Error bars are ± 1SE across 12 participants.

Figure 5 plots the mean SD of the fitted Gaussian curve averaged across participants as a function of stimulus duration for the three display conditions. The lower the SD, the higher the precision of heading judgments. We conducted a 3 (display condition) × 4 (stimulus duration) repeated-measures ANOVA to test for statistical significance of any differences. Across all display conditions, SD decreased (precision increased) with stimulus duration (main effect of stimulus duration, *F*(1.24, 13.6) = 9.02, *p* = 0.0072, *η*^2^ = 0.45). SD also showed a clear difference between display conditions (main effect of display condition, *F*(1.54, 16.90) = 9.85, *p* = 0.0026, *η*^2^ = 0.47). The interaction effect of stimulus duration and display condition was not significant (*F*(1.53, 16.86) = 2.07, *p* = 0.16, *η*^2^ = 0.16).

Newman-Keuls tests revealed that across the four stimulus durations, SD was significantly lower (precision higher) in the drift-only (mean SD ± SE: 1.74° ± 0.14°) than the extra-drift condition (2.86° ± 0.33°, *p* = 0.0018). The addition of target drift to extra-drift cues also produced a significant decrease in SD (increase in precision) in the drift + extra-drift condition (1.58° ± 0.10°) compared with the extra-drift condition (*p* = 0.0015).

### Discussion

The most salient finding of this experiment is that across all four stimulus durations tested, precision with the target drift cue alone is higher than with extra-drift cues alone. Precision with the target drift cue alone is almost identical at 0.4, 0.8, and 1.6 seconds and similar to precision with the combined cues. This indicates a possible ceiling effect in heading judgments with the target drift cue alone starting at the duration of 0.4 second. At the shortest duration of 0.2 second, there is a hint of optimal cue combination: Judgments with the combination of target drift and extra-drift cues are more precise than with either type of cue alone. To explore this possibility, we added an additional measure of performance in the third experiment, reaction time.

## Experiment 3: Measuring reaction time

In this experiment, we replicated the 0.4-second stimulus duration condition of Experiment 2 but asked participants to make heading judgments as quickly as possible. This allowed us to collect parallel measures of reaction times. Although we expected to find the same pattern of the precision data as observed in Experiment 2, the question was whether the addition of extra-drift cues would speed up heading judgments compared with the target drift cue alone. If extra-drift cues reduced reaction time, this would provide evidence of optimal cue combination over longer (>0.2 second) stimulus durations.

### Methods

#### Participants

Twenty participants (11 males, nine females; all naïve as to the purpose of the experiment) between the age of 19 and 36 (average age = 26) at the University of Hong Kong participated in this experiment. Among these participants, four participated in Experiment 1, two in Experiment 2, and two participated in both Experiments 1 and 2. All had normal or corrected to normal vision and provided informed consent. The experiment was approved by the Human Research Ethics Committee for Non-Clinical Faculties at The University of Hong Kong. We determined the sample size based on the observed effect size in Experiment 1.

#### Visual stimuli and procedure

The displays were identical to those in Experiment 2 except that we only tested the stimulus duration of 0.4 second. The testing procedure was the same as that in Experiment 1. The reaction time of heading judgments was measured as the time elapsed from the end of the displayed motion to the mouse click in each trial. Each participant completed 120 trials (8 trials × 15 levels of target-heading angles) in a random order for each display condition. The testing order of the display conditions was counterbalanced between participants. An experiment session typically lasted 30 minutes.

### Results

**Figure 6.**
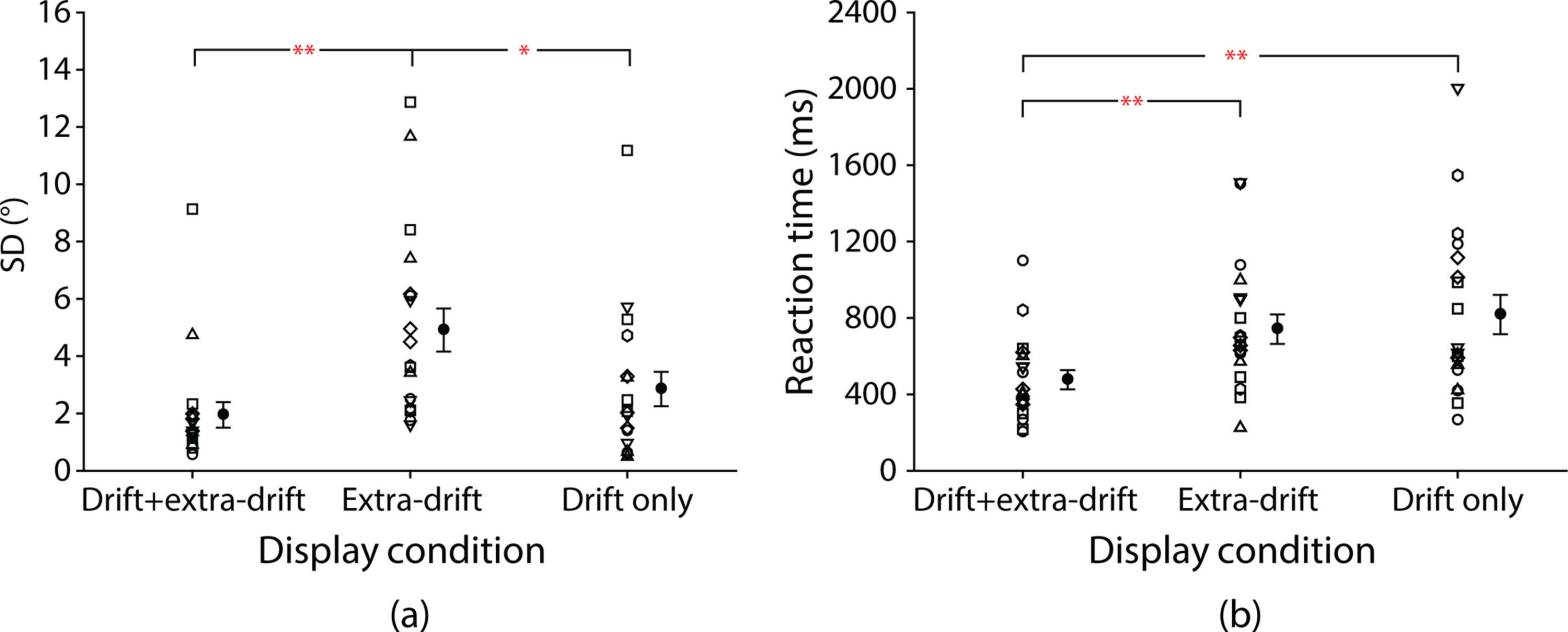
Precision and reaction time for individual participants with group mean shown to the right. (**a**) SD of the fitted Gaussian curve (lower SD indicates higher precision) and (**b**) reaction time of heading judgments against the three display conditions (stimulus duration: 0.4 second). Error bars are ±1SE across 19 participants. *p < 0.05; **p < 0.01.

One participant (female) showed a random pattern of heading judgments that could not be fitted by a cumulative Gaussian function in the drift-only condition. This participant's judgment data were thus excluded from the data analysis. Figure 6 plots SD of the fitted Gaussian curve and reaction time of heading judgments for each individual participant along with the group mean against display condition. The lower the SD, the higher the precision of heading judgments. We conducted two separate one-way repeated-measures ANOVA to test for statistical significance of any differences.

Precision showed a significant difference between display conditions (main effect of display condition: *F*(1.52, 27.27) = 6.70, *p* = 0.0076, *η*^2^ = 0.27). Newman-Keuls tests revealed that SD was significantly lower (precision higher) in the drift-only (mean SD ± SE: 2.85° ± 0.60°) than the extra-drift condition (4.91° ± 0.75°, *p* = 0.018). The addition of target drift to extra-drift cues also significantly decreased SD (increased precision) in the drift + extra-drift condition (1.95° ± 0.45°) compared with the extra-drift condition (*p* = 0.0030). Consistent with what we found at the stimulus duration of 0.4 s in Experiment 2, SD was not higher in the drift + extra-drift than the drift-only condition (*p* = 0.28).

We noted a couple of outliers in each display condition in Figure 6a. To test the robustness of the findings, we repeated the analysis using nonparametric sign tests and found a similar pattern of results to the parametric tests. The medians of SDs for the three display conditions (drift + extra-drift: 1.38°; extra-drift: 3.67°; drift-only: 2.02°) also showed a similar pattern as the means.

Reaction time also showed a significant difference between display conditions (main effect of display condition: *F*(1.80, 32.32) = 8.32, *p* = 0.0017, *η*^2^ = 0.32). Newman-Keuls tests showed that reaction time was not significantly different in the extra-drift (mean ± SE: 757 ± 77 ms) and drift-only conditions (817 ± 103 ms, *p* = 0.50). However, reaction time was significantly shorter in the drift + extra-drift condition (476 ± 51 ms) than the extra-drift (*p* = 0.0035) and the drift-only conditions (*p* = 0.0015).

Again, we noted a couple of outliers in each display condition in Figure 6b. To test the robustness of the findings, we repeated the analysis using nonparametric sign tests and found the same pattern of results to the parametric tests. The pattern of medians of reaction times for the three display conditions (drift + extra-drift: 409 ms; extra-drift: 677 ms; drift-only: 617 ms) also mirrored that of the means.

### Discussion

In this experiment, we looked for evidence of cue-combination. We replicated the 0.4-second condition of Experiment 2 but also measured reaction times. As in the first two experiments we found that precision was higher with the target drift cue alone than with extra-drift cues. Although the highest precision appeared to be in the combined cue condition, the difference in precision between the combined cue and the drift-only cue conditions did not reach statistical significance.

We observed that reaction times were shorter when both the target drift and extra-drift cues were available compared to when each type of cue was presented in isolation. These findings suggest the possibility of an early, optimal combination of cues for heading judgments. However, when we fitted the reaction time data to a race model, which assumes that target drift and extra-drift cues compete and independently determine reaction times, we found that the model predictions were similar to reaction times observed in the combined cue condition (*t*(18) = 0.16, *p* = 0.88, Cohen's *d* = 0.039).

The results of the first three experiments are consistent in showing that heading judgments with the target drift cue alone are typically more precise than with extra-drift cues. In addition, looking across the three experiments, the results are suggestive of the use of both target drift and extra-drift cues for heading judgments when they are both present, and the reaction time data suggests the two cues are processed independently rather than optimally combined. In these experiments, we focused on heading judgments relative to a given object. In the last experiment, we examined heading judgments relative to the entire scene rather than an object of interest.

## Experiment 4: Judgments of heading relative to the scene

In the previous three experiments, participants were asked to make nominal left/right judgments of heading relative to the target. In this experiment, we asked participants to make judgments of absolute heading (i.e., at the end of the trial, participants were asked to use a mouse to move a probe to indicate where they were heading relative to the scene). In addition to the precision of heading judgments, we also measured the accuracy of heading judgments with target drift and extra-drift cues presented in isolation and together to evaluate the contribution of these two types of cues to judgments of absolute heading. We tested heading directions up to 10° to the target to examine whether the effectiveness of the target drift cue changes with the increase of target-heading angle.

### Methods

#### Participants

Twenty students and staff (10 males, 10 females; 19 naïve as to the specific goals of the experiment) between the age of 18 and 41 (average age = 23) at New York University Shanghai and East China Normal University participated in the experiment. All had normal or corrected to normal vision and provided informed consent. The experiment was approved by the Institutional Review Board at New York University Shanghai. We determined the sample size based on the observed effect size in the previous experiments.

#### Visual stimuli and procedure

All three display conditions were tested. The displays were identical to those in Experiment 1 except that 15 regularly spaced initial target-heading angles were tested with the heading direction ranging from −10° (left) to 10° (right) of the target at the beginning of the trial.

The testing procedure was similar to that in Experiment 1 except that at the beginning of each trial, a white fixation cross appeared at the center of a blank screen for one second, and participants were instructed to keep their gaze direction on the cross (i.e., the center of the screen). The fixation cross then disappeared, and a static view of the scene appears for one second to allow participants to fuse the two half-images of the stereo display. The scene then moved for 1.5 seconds to simulate forward movement of the observer. Once the simulated movement stopped, a blue line probe (0.063° H × 0.63° V) appeared in the scene at a randomized position on an invisible arc of a circle centered on the observer's head (see Figure 2d). The radius of the circle was equal to the distance between the observer and the target. Participants were asked to use the mouse to move the probe along the invisible arc to align it with their judged heading direction.

Each participant completed 120 trials (8 trials × 15 levels of target-heading angles) in a random order for each display condition. Participants received 30 randomized training trials (15 levels of target-heading angles × 2 trials) at the beginning of each display condition. No feedback was given on any trial. The testing order of the three display conditions was counterbalanced between participants. An experiment session typically lasted one hour.

### Results

**Figure 7.**
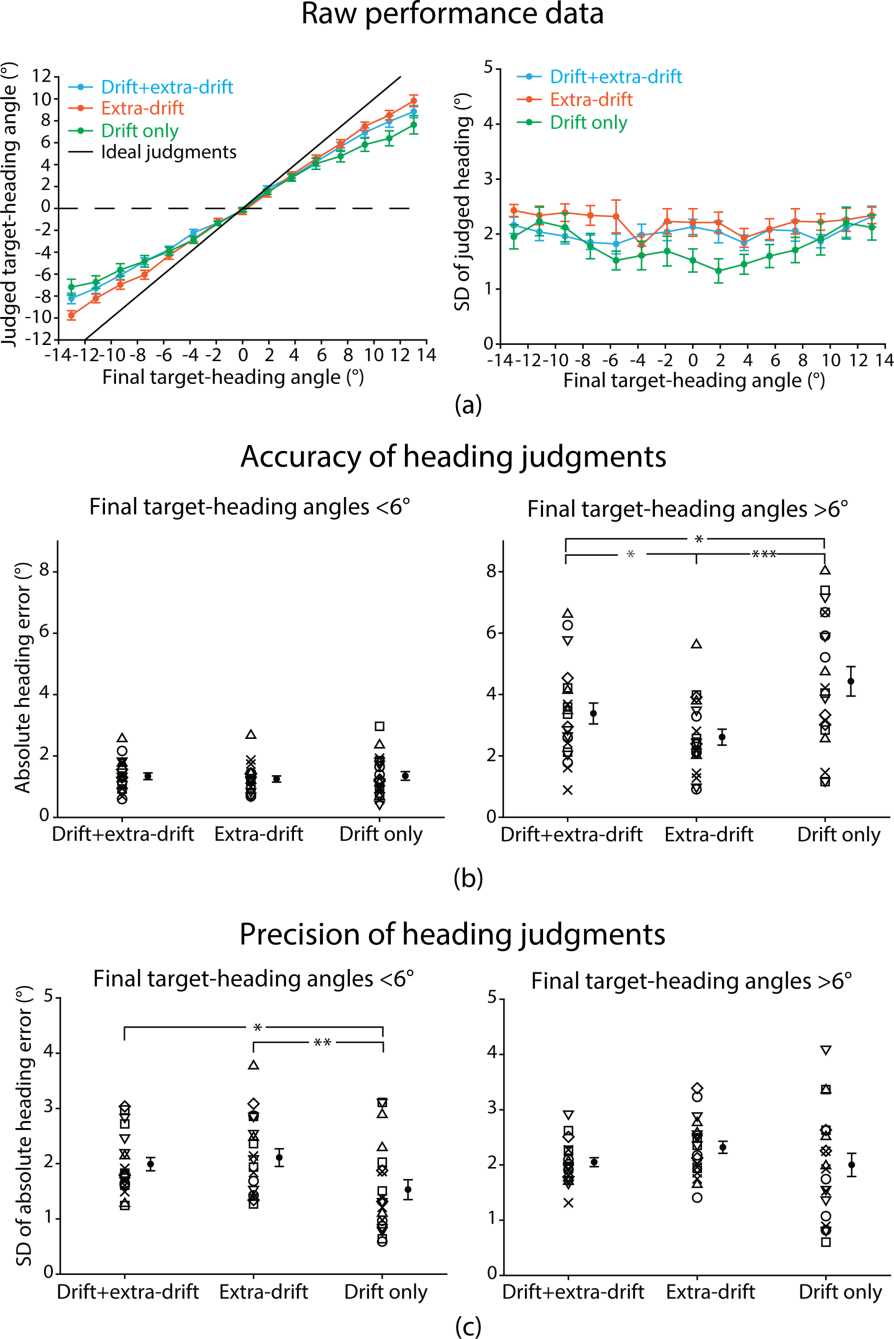
(**a**) Raw performance data. Mean (left panel) and mean SD (right panel) of judged target-heading angle averaged across participants as a function of final target-heading angle for the three display conditions. Lower SD indicates higher precision. (**b**) Mean absolute heading error averaged across final target-heading angles <6° (left) or >6° (right) for each individual participant along with the group mean. (**c**) Mean SD of absolute heading error averaged across final target-heading angles <6° (left) or >6° (right) for each individual participant along with the group mean. Error bars are ± 1SE across 20 participants. *p < 0.1; *p < 0.05; **p < 0.01; ***p < 0.001.

To facilitate the comparison of absolute heading judgments in the current experiment with nominal heading judgments in the previous three experiments, for each participant, we calculated the angle between the judged heading and the target direction at the end of the trial to obtain the judged target-heading angle. Figure 7a plots the mean (left panel) and the SD (right panel) of the judged target-heading angle averaged across participants as a function of the actual final target-heading angle for the three display conditions. For all three display conditions, participants judged heading as being closer to the target than it really was thus displaying a systematic underestimation as reported in previous studies (e.g., Li & Warren, 2000; Sun et al., 2020; Xing & Saunders, 2022). The judged target-heading angles were approximately 50% of the actual final target-heading angles in all three displays conditions for the final target-heading angles within the range of −6° and 6° and then they diverged with a larger underestimation shown in the drift-only than the extra-drift condition. The drift + extra-drift condition appeared to be a weighted average of the other two conditions. However, opposite to the judged target-heading angle data, differences in the SDs of the judged heading angle between the conditions were apparent for the final target-heading angles within the range of −6° and 6°.

To compare the accuracy of heading judgments across the three display conditions, we calculated absolute heading error (i.e., the absolute deviation angle between the judged and the actual final target-heading angle). Figure 7b plots the mean absolute heading error averaged across final target-heading angles <6° (left panel) and >6° (right panel), respectively, against display condition for each individual participant along with the group mean. We conducted two separate one-way repeated-measures ANOVA to test for statistical significance of any differences. For final target-heading angles <6°, accuracy was similar for the three display conditions (main effect of display condition: *F*(1.44, 27.31) = 0.37, *p* = 0.63, *η*^2^ = 0.019). In contrast, for final target-heading angles >6°, accuracy showed a significant difference between display conditions (main effect of display condition: *F*(1.26, 23.85) = 10.25, *p* = 0.0023, *η*^2^ = 0.35). Newman-Keuls tests revealed that accuracy was worse in the drift-only (mean error ± SE: 4.43° ± 0.48°) than the extra-drift condition (2.61° ± 0.26°, *p* < 0.001). The addition of extra-drift cues also significantly increased accuracy in the drift + extra-drift condition (3.38° ± 0.34°) compared with the drift-only condition (*p* = 0.013). This indicates that for final target-heading angles >6°, judgments of heading were less accurate with the target drift cue alone.

To compare the precision of heading judgments across the three display conditions, we calculated the SD of absolute heading error. The lower the SD, the higher the precision of heading judgments. Figure 7c plots the mean SD of absolute heading error averaged across final target-heading angles <6° (left panel) and >6° (right panel), respectively, against display condition for each individual participant along with the group mean. We conducted two separate one-way repeated-measures ANOVA to test for statistical significance of any differences. Opposite to accuracy, for final target-heading angles >6°, SD was similar for the three display conditions (main effect of display condition: *F*(1.43, 27.20) = 1.58, *p* = 0.23, *η*^2^ = 0.077). In contrast, for final target-heading angles <6°, SD showed a significant difference between display conditions (main effect of display condition: *F*(2, 38) = 5.40, *p* = 0.0086, *η*^2^ = 0.22). Newman-Keuls tests revealed that SD was lower (precision higher) in the drift-only (mean SD ± SE: 1.53° ± 0.18°) than the extra-drift condition (2.11° ± 0.16°, *p* = 0.0096). The addition of target drift to extra-drift cues did not significantly decrease SD (increase precision) in the drift + extra-drift condition (1.99° ± 0.12°) compared with the extra-drift condition (*p* = 0.51). This indicates that for final target-heading angles <6°, heading judgments were more precise with the target drift cue alone than with extra-drift cues.

### Discussion

In this experiment, we adopted the method of adjustment and asked participants to make judgments of the heading relative to the entire scene. The results show that when target-heading angle is smaller than 6°, consistent with the findings of the previous three experiments, the precision of judgments is significantly higher with the target drift cue alone than with extra-drift cues (mean SD = 1.53° vs. 2.11°). The accuracy of judgments is comparable across all three display conditions (mean absolute error ≤1.35°). In contrast, when target-heading angle is larger than 6°, the precision of judgments is comparable across all three display conditions (mean SD ≤2.32°). The accuracy of judgments is significantly lower with the target drift cue alone than extra-drift cues (mean absolute error = 4.43° vs. 2.61°), indicating that heading judgments are more accurate with extra-drift cues when heading is not in close proximity to the target.

## General discussion

The results across all four experiments show that the precision of heading judgments is at least as high in the drift-only condition as in the extra-drift condition, thus supporting the claim that the target drift cue can provide precise information about both nominal (Experiments 1–3) and absolute (Experiment 4) direction of heading. The results suggest that the visual system uses both target drift and extra-drift cues for heading judgments when both are present. The results of Experiments 1–3 show that the addition of the drift cue to extra-drift cues increases precision compared to extra-drift cues alone. The reaction time data in Experiment 3 are compatible with the two cues being processed independently. However, although not statically significant, the trend of the precision data at 200 ms in Experiment 2 and at 400 ms in Experiment 3 is also compatible with an early optimal cue combination. Therefore the question of cue-combination in heading judgments warrants further investigation.

In addition to precision, Experiment 4 examined accuracy and found that when heading was in close proximity to the target (<6°), heading judgments were similarly accurate in all three cue conditions. With more eccentric heading directions (>6°), accuracy reduced in all three conditions and reduced most in the drift-only condition. Interestingly, a similar relationship between eccentricity and inaccuracy has been reported in judgments of the direction of approaching objects (Harris & Dean, 2003), a task that is comparable to judging heading in the drift-only condition in the current study.

### Possible confounds?

Is it possible that participants did not perform the task as we anticipated but instead used a heuristic to infer heading from the two-dimensional image motion of the target on the display screen? That is: The target drifted leftward, therefore I must be moving to the right and vice versa. This is very unlikely for the following reasons. First, participants received no feedback, so they would not know this was an appropriate strategy to pursue. Second, no participant reported doing this during the debrief. Third, using such an artificial strategy for nominal left/right heading judgments involves a conscious reverse mapping (i.e., target moved left, I press the right button, and vice versa). This would impose a time penalty, leading to longer reaction times with the target drift cue alone than with extra-drift cues. We observed no such effect in Experiment 3. Last, although the direction of target drift on the screen indicates heading relative to the target, such a cue does not indicate heading relative to the entire scene. Without a percept of heading, participants would not be able to make accurate and precise judgments of heading relative to the scene as observed in Experiment 4.

### Relationship of our study to Llewellyn (1971) and Wilkie and Wann (2003)

Llewellyn (1971) was the first to investigate a potential role for target drift, a cue previously identified by Rock (1966), in the visual guidance of locomotion. Llewellyn did not address the question of whether drift *does* contribute to judgments of heading or the guidance of locomotion, but he did establish that it *could* be used, that humans are sufficiently sensitive to the cue. He examined observers’ sensitivity to target drift by measuring detection latency when the target object was presented in isolation or against a background of other scene elements. Interestingly, he found that the detection latency was shorter when target drift was presented in isolation. He also examined observers’ precision in cancelling target drift and compared it to the precision in indicating the FoE in an optic flow pattern. On the basis of 10 experiments, he concluded that target drift, not optic flow, is the key cue used in the visual guidance of locomotion.

Since Llewellyn's publication, as explained in the introduction, the focus of research has been on establishing the potential utility of other cues, optic flow in particular; target drift has not been studied since. The single exception we have been able to identify is Experiment 1 in Wilkie and Wann (2003). In their experiment, the authors perturbed what they called the “ER” (extra-retinal) cue by rotating the entire display, including the viewing frame, around the observer. This manipulation put the target drift cue in conflict with extra-drift cues. The authors reported the rotation had an impact on the accuracy of judgments of linear heading direction. The authors did not describe their findings in terms of Llewellyn's target drift, but their finding is compatible with its use in judgments of heading.

In the current study, across four experiments, we have demonstrated the use of target drift as a cue to heading judgments. Target drift is usually removed in laboratory experiments, but in natural circumstances when it is normalized with cues to motion-in-depth (see Figure 2), our data show that target drift can support remarkably precise judgments of both nominal heading relative to a target and absolute heading relative to the entire scene. It is thus clear that target drift is a powerful cue for heading judgments. This highlights the significance of Llewellyn's work and suggests that after 50 years of neglect, it is timely for the field to revisit the role of target drift as a cue to visual guidance of locomotion. Indeed, Rushton and Allison (2013) showed that it is possible to steer to a target using the target drift cue alone. Their model used a proportional controller to reduce drift to zero. After each step, the actor (robot or animal) would turn by an amount that was proportional to the magnitude of the drift. When the proportion was held at 1, the actor would follow an equiangular spiral path to the target; when the proportion was held at a value >1, the path would home in on a straight-line path.

### Advantages and disadvantages of different visual cues to judgments of heading

It is important to note that the usefulness of target drift and extra-drift cues to judgments of heading depends on not only the type of environment but also the distance of the target and scene objects. For example, splay angle is only useful when there are path edges, and perspective shape cues are only useful when there are large planar surfaces. Although the egocentric direction of the target is always useful for guiding walking irrespective of the distance of the target or the rest of the scene, target drift is only informative about the direction of heading when the target object is reasonably close. When the target object is far, target drift-only indicates the change in orientation of the observer relative to the target, not the direction of heading relative to the target. (Note that “close” and “far” here refer to relative distance in time, i.e., time to contact or time-to-passage. When the observer is travelling fast, target drift may be salient even when the target is far in absolute distance.) In contrast, optic flow and differential motion parallax can be informative about heading relative to a distant object, irrespective of the distance of the target, provided there are some close scene objects in view.

It is thus not helpful to ask which cue is the “dominant” cue in judgments of heading, but rather to recognize that the contribution of different cues likely depends on the content of the environment and the task. The challenge is to identify which circumstances favor which cues and how the cues are combined. Cutting and Vishton's (1995) analysis of depth cues provides an excellent model for this.

Note that throughout this paper, we have chosen to use the term “heading judgments” rather than the commonly used term “heading perception”. The reason for this is twofold: The information in the changing optic array that provides information about heading is also used by other animals and insects (e.g., Bhagavatula, Claudianos, Ibbotson, & Srinivasan, 2011; Collett, 2002; Srinivasan, Zhang, Lehrer, & Collett, 1996), and robots and computer vision systems (see Serres & Ruffier, 2017 for a review). It seems strange to talk about “heading perception” when referring to insects or computer vision system, and confusing to use different terms for the same process (heading perception vs. heading judgments) just because humans have an accompanying phenomenal experience.

### Summary

Despite its potential significance, target drift as a cue for heading direction has been largely overlooked in the past 50 years of research on heading perception. In this study, we investigated the effectiveness of target drift as a cue for heading judgments. Across four experiments, three focusing on target-relative judgments (nominal) and one on scene-relative judgments (absolute), we found that heading judgments based on target drift alone can be at least as precise as those relying on extra-drift cues. We hope these findings will prompt further research to examine the utility of the target drift cue and how it can be combined with other cues in heading judgments.

## Footnotes

1 Hoyle's novel, “The Black Cloud” is often cited as the root of work on estimation of time-to-contact. Interestingly in the same book, Hoyle describes how target drift and relative target drift rate can be used to estimate the direction of relative movement between an object and an observation point (see Rushton & Gray, 2006).

2 We also tested 0.1-second duration, but only a few participants could generate reliable data at this stimulus duration. The data of 0.1-second duration were thus not included in the data analysis.
